# *Dirofilaria immitis* JYD-34 isolate: whole genome analysis

**DOI:** 10.1186/s13071-017-2437-5

**Published:** 2017-11-09

**Authors:** Catherine Bourguinat, Francois Lefebvre, Johanna Sandoval, Brenda Bondesen, Yovany Moreno, Roger K. Prichard

**Affiliations:** 1 Institute of Parasitology, McGill University, 21, 111 Lakeshore Road, Sainte Anne de Bellevue, QC H9X3V9 Canada; 2 Canadian Centre for Computational Genomics, McGill University and Genome Quebec Innovation Centre, Montreal, QC Canada; 3 Pharma Discovery & Research, Merial Inc, Duluth, GA USA

**Keywords:** *Dirofilaria immitis*, JYD isolate, Heartworm preventives, Macrocyclic lactone resistance, Whole genome analysis

## Abstract

**Background:**

Macrocyclic lactone (ML) anthelmintics are used for chemoprophylaxis for heartworm infection in dogs and cats. Cases of dogs becoming infected with heartworms, despite apparent compliance to recommended chemoprophylaxis with approved preventives, has led to such cases being considered as suspected lack of efficacy (LOE). Recently, microfilariae collected from a small number of LOE isolates were used as a source of infection of new host dogs and confirmed to have reduced susceptibility to ML in controlled efficacy studies using L3 challenge in dogs. A specific *Dirofilaria immitis* laboratory isolate named JYD-34 has also been confirmed to have less than 100% susceptibility to ML-based preventives. For preventive claims against heartworm disease, evidence of 100% efficacy is required by FDA-CVM. It was therefore of interest to determine whether JYD-34 has a genetic profile similar to other documented LOE and confirmed reduced susceptibility isolates or has a genetic profile similar to known ML-susceptible isolates.

**Methods:**

In this study, the 90Mbp whole genome of the JYD-34 strain was sequenced. This genome was compared using bioinformatics tools to pooled whole genomes of four well-characterized susceptible *D. immitis* populations, one susceptible Missouri laboratory isolate, as well as the pooled whole genomes of four LOE *D. immitis* populations. Fixation indexes (F_ST_), which allow the genetic structure of each population (isolate) to be compared at the level of single nucleotide polymorphisms (SNP) across the genome, have been calculated. Forty-one previously reported SNP, that appeared to differentiate between susceptible and LOE and confirmed reduced susceptibility isolates, were also investigated in the JYD-34 isolate.

**Results:**

The F_ST_ analysis, and the analysis of the 41 SNP that appeared to differentiate reduced susceptibility from fully susceptible isolates, confirmed that the JYD-34 isolate has a genome similar to previously investigated LOE isolates, and isolates confirmed to have reduced susceptibility, and to be dissimilar to the susceptible isolates.

**Conclusions:**

These results provide additional evidence for the link between genotype and the reduced susceptibility phenotype observed in such isolates as JYD-34. Further work on other isolates showing reduced susceptibility to ML is required to demonstrate the value of genetic analysis in predicting the response to ML chemoprophylaxis. The authors suggest that genetic analysis may be useful in helping to interpret the results of in vivo efficacy testing of ML heartworm preventives against *D. immitis* isolates.

**Electronic supplementary material:**

The online version of this article (10.1186/s13071-017-2437-5) contains supplementary material, which is available to authorized users.

## Background

*Dirofilaria immitis* is the causative agent of heartworm disease in dogs and cats [1]. Macrocyclic lactone (ML) anthelmintics are used for chemoprophylaxis against heartworm infection in companion animals [2]. Cases of dogs becoming infected, despite apparent compliance to recommended chemoprophylaxis with approved preventives, has led to suspected lack of efficacy (LOE) [3]. Recently, microfilariae (MF) collected from a small number of LOE isolates, after development in mosquitoes to L3 larvae, were used to infect new host dogs and the isolates confirmed to be resistant to ML prophylaxis in controlled efficacy studies in dogs challenged with the L3 larvae [4, 5]. *D. immitis* JYD-34 was originally isolated from the field and taken into the laboratory by TRS Labs Inc. (Athens, Georgia, USA) where it was subsequently found to have less than 100% susceptibility to 3 ML-based preventives [5, 6]. For claims for prevention of heartworm disease, evidence of 100% efficacy is required by the US Food and Drug Administration (https://www.fda.gov/ucm/groups/fdagov-public/@fdagov-av-gen/documents/document/ucm052652.pdf). In previous studies [5], it was found that a number of LOE isolates and isolates confirmed in efficacy studies to be resistant, had different genetic profiles to susceptible isolates. It was therefore of interest to determine whether JYD-34 has a genetic profile similar to the LOE and resistant isolates previously analyzed or has a genetic profile similar to the previously analyzed ML-susceptible isolates [5].

## Methods

### Samples – DNA extraction – Sequencing

The JYD-34 pool of MF was provided by TRS Labs Inc. (Athens, Georgia, USA). JYD-34 *D. immitis* was originally isolated in 2010 in a heartworm-positive dog from Illinois. The original dog had no known history of treatment with ML products. *D. immitis* MF were purified from whole canine blood using a protocol previously described [7]. DNA was isolated using the DNeasy extraction kit (Qiagen) following the manufacturer’s instructions. DNA integrity was verified by electrophoresis on a 0.8% agarose gel, and its purity was assessed by measuring the OD ratios at 260/280 nm and 260/230 nm. Frozen DNA was shipped to the Beijing Genomics Institute (www.bgi.com) for whole genome sequencing. DNA was then fragmented randomly. After electrophoresis, DNA fragments of desired length were gel purified. Adapter ligation and DNA cluster preparation were performed and subjected to Solexa sequencing [8–10] for next- generation sequencing using the Illumina HiSeq™ 2000. To minimize the likelihood of systematic bias in sampling, two paired-end libraries of the same DNA pool sample with insert size of 500 bp were prepared and were then subjected to whole-genome sequencing to generate 90-bp-paired-end reads. The four FASTQ files generated were sent to McGill University for analysis.

### BAM file for JYD-34

Reads were trimmed from the 3-prime-end to generate a Phred quality score [11, 12] of at least 30. Illumina sequencing adapters were removed from the reads, and all trimmed reads were required to have a length of at least 50 bp. Trimming and clipping were performed using Trimmomatic software (http://www.usadellab.org/cms/?page=trimmomatic) [13]. Any DNA read from *Canis familiaris* were removed from the data. The filtered reads were aligned to the nDi.2.2.*D. immitis* genome (http://www.nematodes.org/genomes/dirofilaria_immitis/). Each readset was aligned using BWA (http://bio-bwa.sourceforge.net/) [14], which created a Binary Alignment Map file (BAM).

### Comparison of genomes between different *D. immitis* isolates

PoPoolation 2, adapted for analysis of pooled samples [15, 16], was used. A mpileup file was generated with a minimum quality score of Q20 using BAM files from the JYD-34 genome, and from susceptible and LOE isolates from Bourguinat et al. (2015) that included data pooled from four susceptible isolates (Missouri laboratory isolate, maintained at TRS Labs since 2000; Gran Canaria field isolate; Grenada field isolate; Italy field isolate) and from four LOE field isolates (Mechanicsville [Virginia], New Orleans [Louisiana], Haywood County [Tennessee] and Monroe [Louisiana]) and separately from the susceptible Missouri laboratory isolate (from TRS Labs). A subsequent synchronized file was generated following PoPoolation 2 directives. F_ST_ or fixative index was calculated on each single nucleotide polymorphism (SNP) in the genome, based on the synchronized file. The criteria for F_ST_ calculation were set with a minimum nucleotide count of six, a minimum and maximum read coverage of 30 and 10,000, respectively. The distance between two populations (Susceptible versus JYD-34, Susceptible versus LOE, Missouri versus JYD-34, Missouri versus LOE and JYD-34 versus LOE) was calculated as the mean F_ST_ value for all SNPs. Clustering was assessed based on filtered SNPs using various minimal F_ST_ thresholds ranging from 0 to 0.9 where F_ST_ = 0 means no divergence between two population and F_ST_ = 1 complete divergence. Dendrograms were built to visualize distance between populations using R (https://www.r-project.org/) and F_ST_ means as distances.

### Comparison of *D. immitis* populations using SNP previously reported

Forty-one SNP previously reported [5] were investigated. The program BVA Tools (https://bitbucket.org/mugqic/bvatools/src) was used to extract, from the JYD-34 BAM file, the nucleotide counts at each of the 41 SNP of interest. The default quality score used was Q10. The nucleotide counts were assimilated to the allele counts, and allele frequencies were calculated. Allele frequencies for the susceptible (SUS), LOE and resistant (RES) populations were retrieved from the genotype frequencies published [5].

## Results

### Comparison of genomes between different *D. immitis* isolates

Ninety-four percent of the JYD-34 genome had a depth of read sequencing of ≥50×, which was similar to the data available for the Missouri isolate genome (TRS) and for the pooled genomes of susceptible isolates. In comparison, 81 % of the LOE genomes had a depth of read sequencing of ≥30×.

**Fig. 1:**
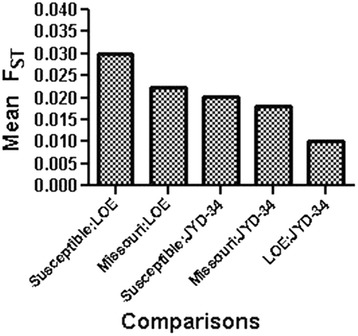
Means F_ST_ calculated from 1,602,214 SNPs over the whole *D. immitis* nDi.2.2 genome, for comparisons between isolates: Susceptible versus JYD-34, Susceptible versus LOE, Missouri versus JYD-34, Missouri versus LOE, and JYD-34 versus LOE. F_ST_ = 0 means no divergence between two populations and F_ST_ = 1 means complete divergence

**Fig. 2:**
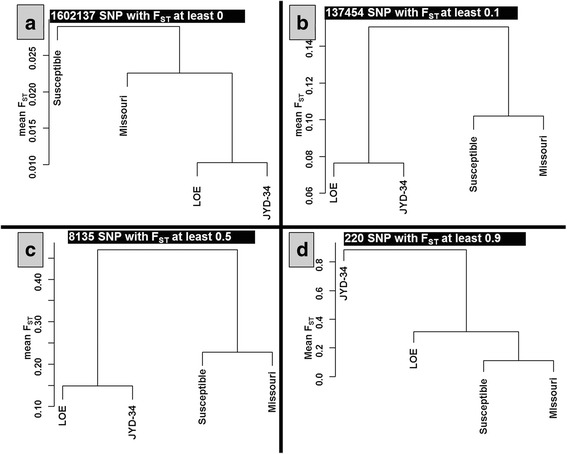
Dendrogram visualization based on mean F_ST_ and different level of F_ST_ threshold (0, 0.1, 0.5 and 0.9 (panels **a**, **b**, **c** and **d**, respectively). The dendrograms illustrate the divergence that exists between JYD-34 isolate and, respectively, Missouri (susceptible laboratory isolate), pooled susceptible and pooled LOE isolates; where F_ST_ = 0 means no divergence between two populations, and F_ST_ = 1 means complete divergence. Additional dendrograms and information are presented in Additional file 1

Based on the criteria used, F_ST_ values were calculated for 1,602,214 SNP over the whole nDi.2.2.*D. immitis* genome (~90 Mb), for each possible comparison between populations. Means F_ST_ values between populations are illustrated in Fig. 1. The divergence between Susceptible and LOE populations was the highest (F_ST_ mean = 0.03). Divergence was also observed between susceptible populations and the JYD-34 isolate, as well as between the Missouri (susceptible) isolate and the JYD-34 isolate. Interestingly, population divergence was the lowest between the LOE populations and the JYD-34 isolate (F_ST_ mean = 0.01). When thresholds for F_ST_ values were stringently set, we were able to understand better the genomic divergence between the populations. The dendrograms that represented the mean F_ST_ distance matrix with 10 different F_ST_ thresholds are illustrated in Additional file 1, while some examples are provided in Fig. 2. The dendograms revealed that the JYD-34 isolate had a genetic profile similar to LOE.

### Comparison of isolates using SNP previously reported

**Fig. 3:**
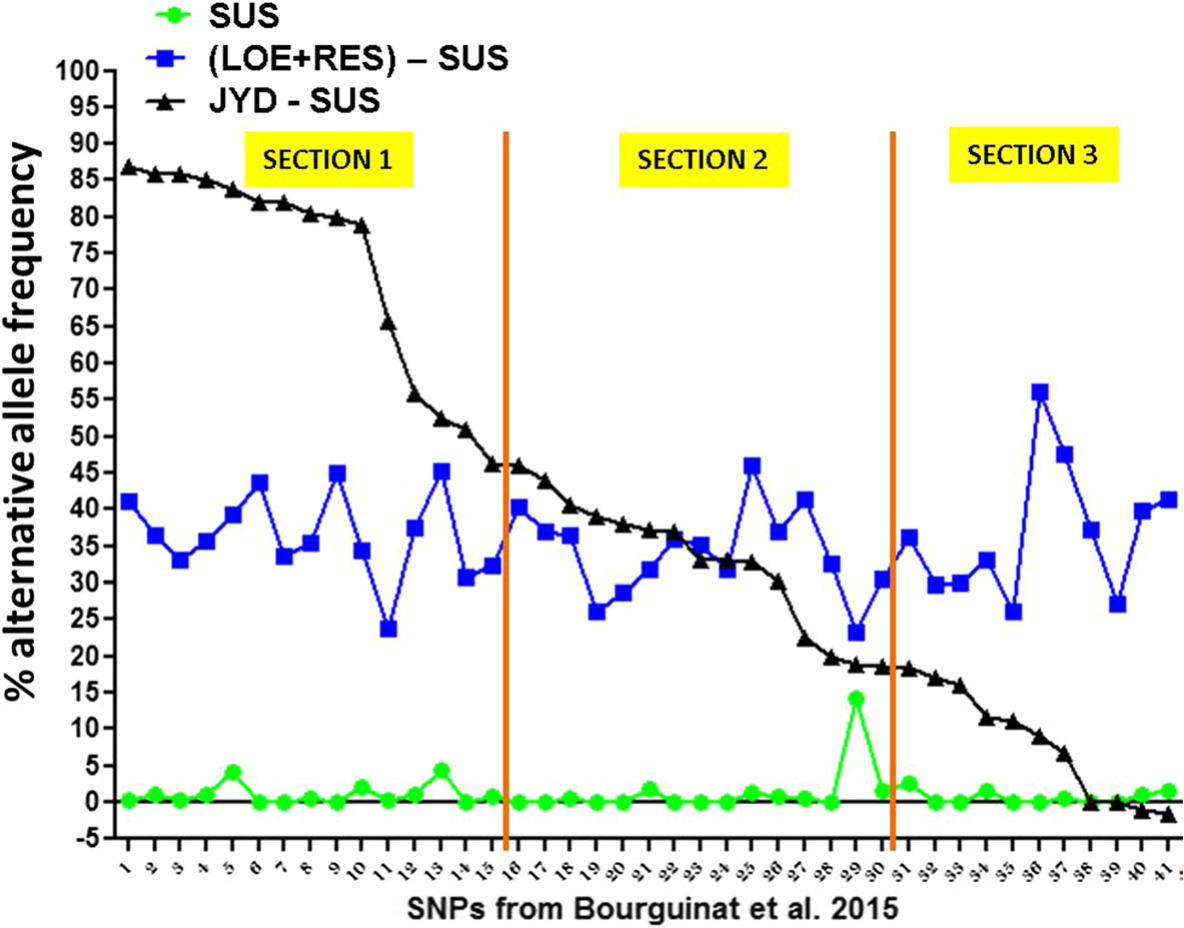
Comparison of the genetic profile of JYD-34 isolate with SUS and (LOE + RES) isolates based on 41 SNP previously reported. Difference in the percentage of the alternative nucleotide frequencies of the 41 alternative nucleotides between the (LOE + RES) isolates and the SUS isolates (blue square shape and line) and between JYD-34 isolate and the SUS isolates (black triangle shape and line) are illustrated. Green round shape and line represent the percentage of the alternative nucleotide frequencies in the SUS isolates. The information of the SNP on the x axis is provided in Additional file 2

In Fig. 3 (and Additional file 2) are summarized the differences in the percentage of the alternative nucleotide frequencies of the 41 alternative nucleotides between the (LOE + RES) isolates and the SUS isolates and between the JYD-34 isolate and the SUS isolates. Interestingly, three distinct sections can be described in Fig. 3; in section 1, the JYD-34 isolate carried higher frequencies of the alternative alleles compared to the (LOE + RES) isolates in 15 SNP; in section 2, JYD-34 isolate had a similar genetic profile compared to (LOE + RES) in 15 SNP; in section 3, 11 SNPs in JYD-34 had a lower percentage frequency of alternative allele compared to the (LOE + RES) isolates and of those four SNP had similar frequencies of the alternative allele in JYD-34 to SUS. While SNP in section 3 are unlikely to differentiate reliably between resistant and susceptible, SNP in section 1 may be the best for differentiating between resistant and susceptible populations.

## Discussion and conclusions

This study provides further evidence that the ML-resistant isolate of *D. immitis*, JYD-34, has an overall genetic profile similar to other isolates that have been described as LOE or confirmed as having reduced sensitivity to ML heartworm preventives [5] and that all of these populations showed different overall genetic profiles compared with known susceptible isolates [5]. While the number of isolates that have so far been subjected to whole genome analysis and comparisons is small, the results to date do suggest a pattern in which isolates that are resistant to ML heartworm preventives have distinct genetic profiles when compared to susceptible isolates. More confirmed susceptible and resistant isolates do need to be investigated to confirm these different profiles. Nevertheless, it is beginning to seem feasible to characterize *D. immitis* isolates as to whether they are likely to be fully susceptible or possibly will show reduced susceptibility to ML heartworm preventives. This could have a number of advantages in terms of maintenance costs for new isolates from the field, unnecessary sacrifice of experimental animals, and time and costs required to determine whether an isolate is likely to respond to ML heartworm preventives as susceptible or resistant. It could also better delineate where possible ML resistance is occurring and be a tool to help reduce the spread of ML resistance.

Ultimately, to manage heartworm disease prevention and control when resistance may be emerging, new tools are needed. Being able to detect likely resistant populations and measure any new therapeutics that may emerge against those populations may be critically important to maintaining high-level control. It will also be important to be able to measure any change in the level of susceptibility over time and to differentiate between real resistance and an inaccurately diagnosed LOE, which should be ascribed to a lack of full compliance with use of heartworm preventives.

Based on whole genome and F_ST_ analysis, the JYD-34 isolate is genetically closer to the LOE and confirmed resistant isolates than to the susceptible isolates so far analyzed. The analysis of the 41 SNP previously reported to possibly differentiate between susceptible and LOE/RES showed that a higher genetic divergence of JYD with SUS existed compared with LOE/RES. This provides additional evidence that the JYD-34 isolate is resistant, and the genetic analysis is consistent with the results found in efficacy studies with this isolate. The JYD-34 isolate could be an ideal isolate to test new molecules and/or products with new modes of action that may have the property of breaking ML resistance in *D. immitis*. Such new antiparasitic drugs would be very desirable should control of heartworm disease not be maintained with ML. An ability to categorize isolates as to whether they are susceptible or resistant to ML preventives may be helpful for the long-term sustainability of highly effective heartworm prevention.

**Fig. 4:**
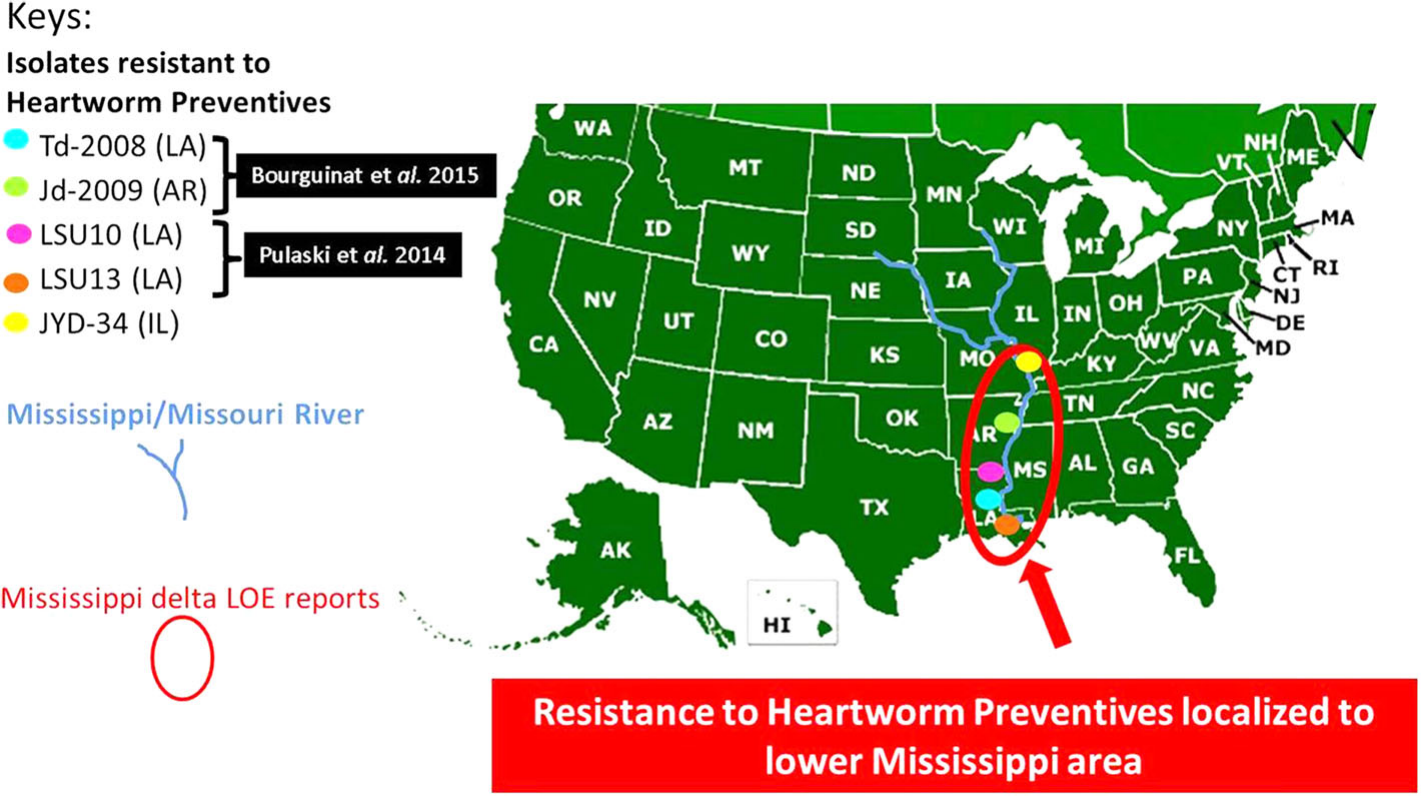
Summary of locations where confirmed resistant isolates to heartworm preventives were reported

Genetic analyses can be valuable and may predict ML response. This analysis suggests that some of the 41 SNP previously identified as possibly useful as markers for reduced efficacy of ML heartworm preventives may not be reliable with all isolates that show reduced susceptibility. On the other hand, many of these SNP have held up as probably being useful for monitoring for reduced susceptibility. However, additional isolates and investigations are needed to increase confidence in a small suite of genetic markers. In that context, some additional results can be found in another study reported in this issue [17]; and further investigation of these markers is underway in order to better define a small subset of markers that can be used to monitor for ML resistance with confidence. Such markers could be used to undertake large-scale surveys for ML resistance in domestic dogs and wild canids. Based on experience with other parasite species in which anthelmintic resistance has been confirmed, there may be an advantage in characterizing isolates as to whether they are genetically susceptible or resistant before efficacy studies are undertaken in vivo, which involves the sacrifice of experimental animals and considerable cost. The current reported resistant isolates are mostly localized in the Mississippi basin (Fig. 4). However, the use of genetic markers may be particularly helpful in better delineating where possible ML preventive resistance could be a threat.

## Additional files

Additional file 1: Dendrogram visualization based on means F_ST._ The dendrograms illustrate the divergence that exists between JYD-34 isolate and Missouri, Susceptible and LOE isolates, where F_ST_ = 0 means no divergence between two population and F_ST_ = 1 means complete divergence. In this analysis, for each SNP out of the total 1,602,137 SNP, the F_ST_ corresponding to each of the different comparisons – LOE/Susceptible, LOE/TRS, LOE/JYD, Susceptible/TRS, Susceptible/JYD and TRS/JYD – were calculated. If at least one of these comparisons had an F_ST_ value of 0.1 or greater, the corresponding SNP and the F_ST_ for each population comparison were retained in the analysis. Based on this criterion, 137,454 SNPs were kept to calculate the mean F_ST_ used in the distance matrix that allowed the construction of the dendrogram. Thresholds were progressively increased from 0 to 0.9. (PDF 228 kb)

Additional file 2: Additional information for Fig. 3 regarding the SNP locations in the nDi.2.2.*Dirofilaria immitis* genome. (XLSX 12 kb)

## Abbreviations

F_ST_: Fixative index
LOE: Lack of efficacy
MF: Microfilaria/e
ML: Macrocyclic lactone
RES: Resistant
SNP: Single nucleotide polymorphisms
SUS: Susceptible
