# Supplementary material for: Dirofilaria immitis JYD-34 isolate: whole genome analysis
Source: Parasit Vectors. 2017 Nov 9;10(Suppl 2):494. doi: 10.1186/s13071-017-2437-5 (PMC5688507; doi:10.1186/s13071-017-2437-5)
Supplement: Supplementary file 1 — Dendrogram visualization based on means FST. The dendrograms illustrate the divergence that exists between JYD-34 isolate and Missouri, Susceptible and LOE isolates, where FST = 0 means no divergence between two population and FST = 1 means complete divergence. In this analysis, for each SNP out of the total 1,602,137 SNP, the FST corresponding to each of the different comparisons – LOE/Susceptible, LOE/TRS, LOE/JYD, Susceptible/TRS, Susceptible/JYD and TRS/JYD – were calculated. If at least one of these comparisons had an FST value of 0.1 or greater, the corresponding SNP and the FST for each population comparison were retained in the analysis. Based on this criterion, 137,454 SNPs were kept to calculate the mean FST used in the distance matrix that allowed the construction of the dendrogram. Thresholds were progressively increased from 0 to 0.9. (PDF 228 kb) [file 13071_2017_2437_MOESM1_ESM.pdf]

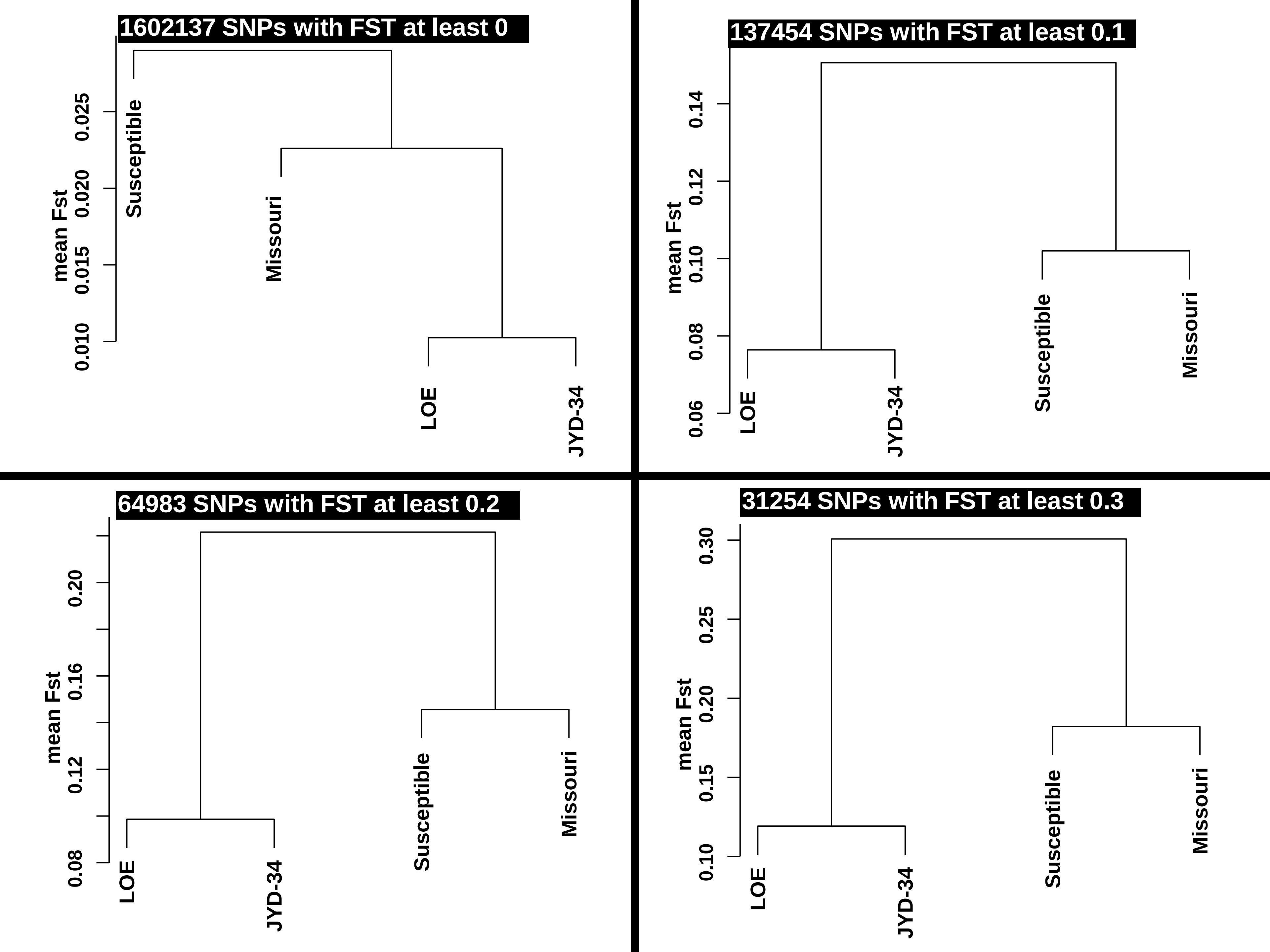

1602137 SNPs with FST at least 0
mean Fst
0.010
0.015
0.020
0.025
Susceptible
Missouri
LOE
JYD-34
137454 SNPs with FST at least 0.1
mean Fst
0.06
0.08
0.10
0.12
0.14
LOE
JYD-34
Susceptible
Missouri
64983 SNPs with FST at least 0.2
mean Fst
0.08
0.12
0.16
0.20
LOE
JYD-34
Susceptible
Missouri
31254 SNPs with FST at least 0.3
mean Fst
0.10
0.15
0.20
0.25
0.30
LOE
JYD-34
Susceptible
Missouri

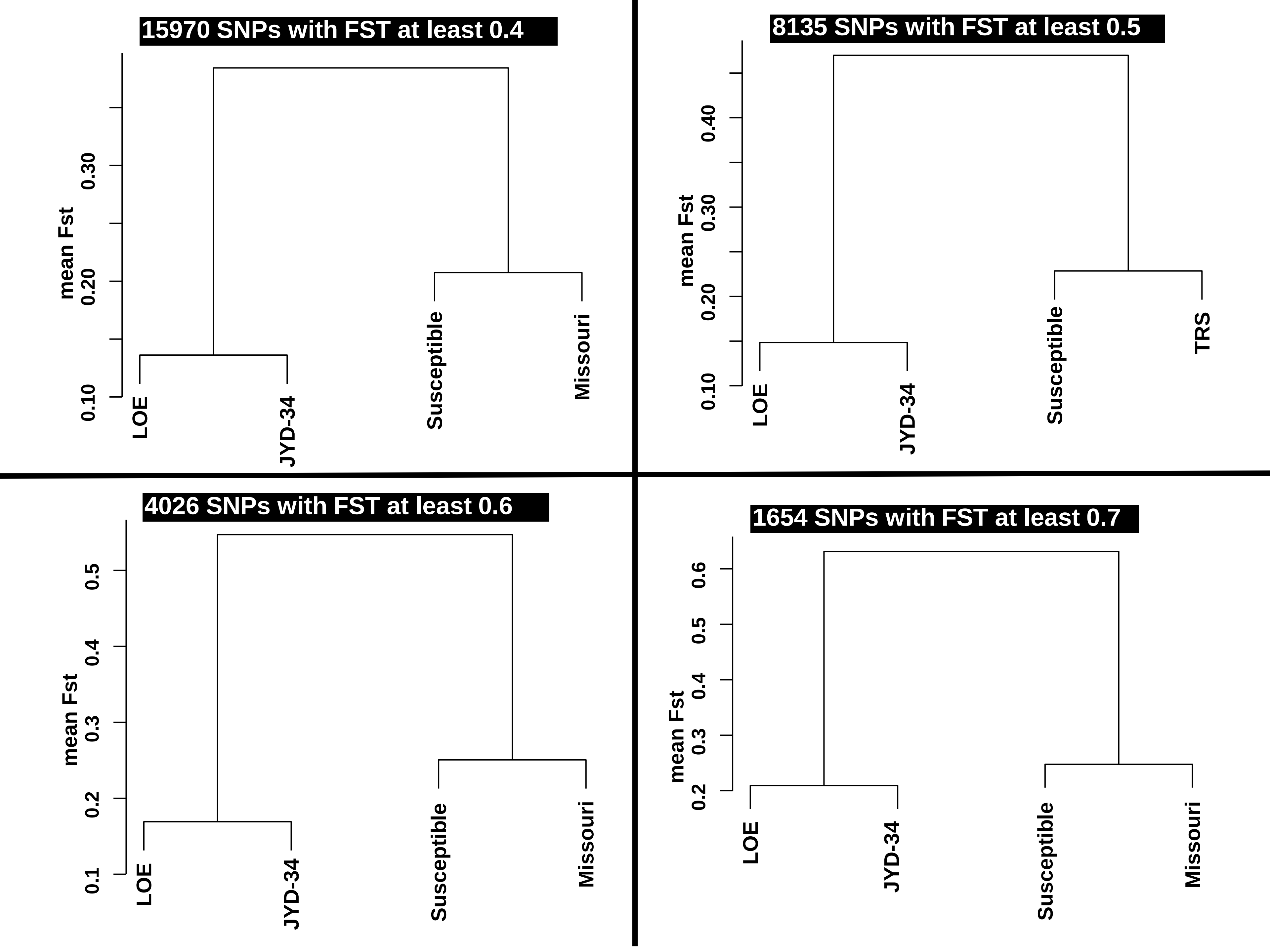

15970 SNPs with FST at least 0.4
mean Fst
0.10
0.20
0.30
LOE
JYD-34
Susceptible
Missouri
8135 SNPs with FST at least 0.5
mean Fst
0.10
0.20
0.30
0.40
LOE
JYD-34
Susceptible
TRS
4026 SNPs with FST at least 0.6
mean Fst
0.1
0.2
0.3
0.4
0.5
LOE
JYD-34
Susceptible
Missouri
1654 SNPs with FST at least 0.7
mean Fst
0.2
0.3
0.4
0.5
0.6
LOE
JYD-34
Susceptible
Missouri

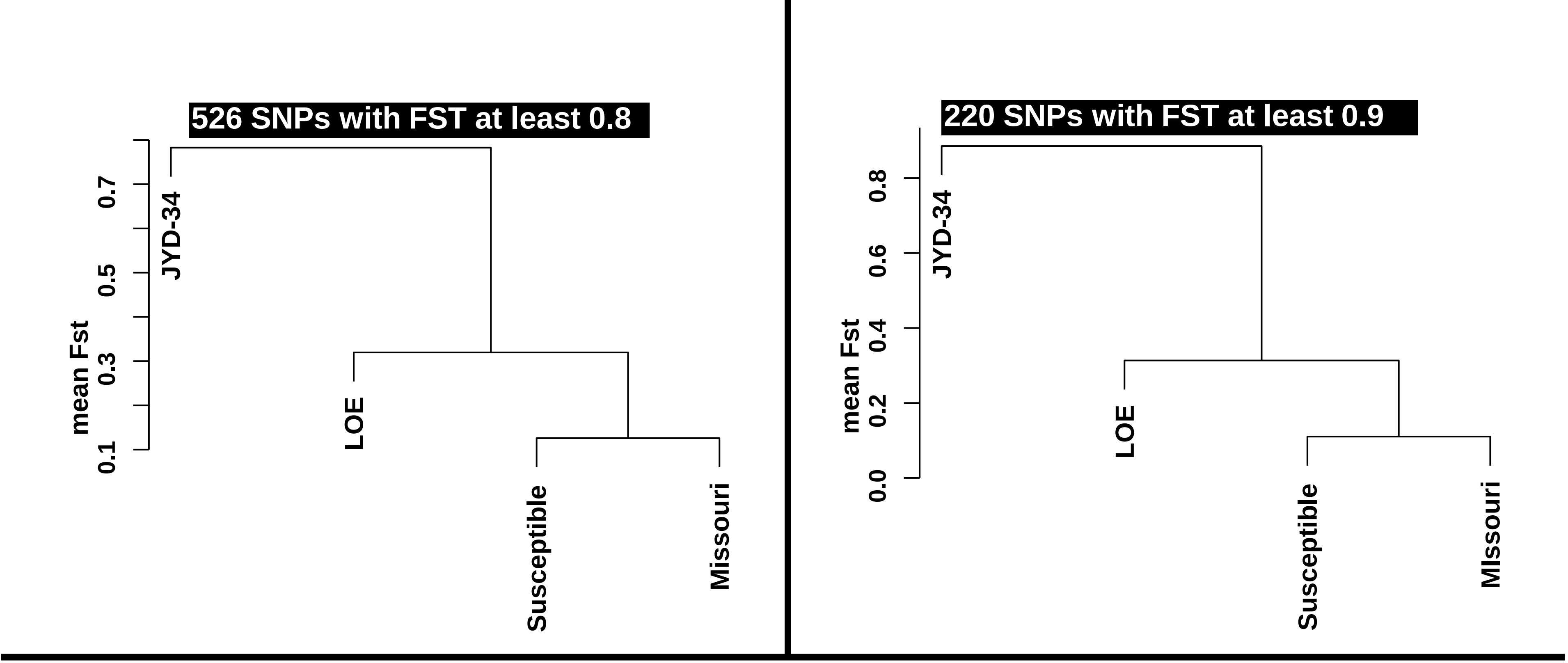

Additional file 1: Dendrogram visualization based on means F_{ST}. The dendrograms illustrate the divergence that exists between JYD-34 isolate and Missouri, Susceptible and LOE isolates, where FST = 0 means no divergence between two population and FST = 1 means complete divergence. In this analysis, for each SNP out of the total 1,602,137 SNPs, the F_{ST} corresponding to each of the different comparisons LOE/Susceptible; LOE/TRS; LOE/JYD; Susceptible/TRS; Susceptible/JYD; and TRS/JYD were calculated. If at least one of these comparisons had an F_{ST} value of 0.1 or greater, the corresponding SNP and the F_{ST} for each population comparison were retained in the analysis. Based on this criterion, 137,454 SNPs were kept to calculate the mean F_{ST} used in the distance matrix that allowed the construction of the dendrogram. Thresholds were progressively increased from 0 to 0.9.
